# Cholecystokinin Elevates Mouse Plasma Lipids

**DOI:** 10.1371/journal.pone.0051011

**Published:** 2012-12-21

**Authors:** Lichun Zhou, Hong Yang, Xinghua Lin, Emmanuel U. Okoro, Zhongmao Guo

**Affiliations:** Department of Physiology, Meharry Medical College, Nashville, Tennessee, United States of America; St. Joseph's Hospital and Medical Center, United States of America

## Abstract

Cholecystokinin (CCK) is a peptide hormone that induces bile release into the intestinal lumen which in turn aids in fat digestion and absorption in the intestine. While excretion of bile acids and cholesterol into the feces eliminates cholesterol from the body, this report examined the effect of CCK on increasing plasma cholesterol and triglycerides in mice. Our data demonstrated that intravenous injection of [Thr28, Nle31]-CCK at a dose of 50 ng/kg significantly increased plasma triglyceride and cholesterol levels by 22 and 31%, respectively, in fasting low-density lipoprotein receptor knockout (*LDLR^−/−^*) mice. The same dose of [Thr28, Nle31]-CCK induced 6 and 13% increases in plasma triglyceride and cholesterol, respectively, in wild-type mice. However, these particular before and after CCK treatment values did not achieve statistical significance. Oral feeding of olive oil further elevated plasma triglycerides, but did not alter plasma cholesterol levels in CCK-treated mice. The increased plasma cholesterol in CCK-treated mice was distributed in very-low, low and high density lipoproteins (VLDL, LDL and HDL) with less of an increase in HDL. Correspondingly, the plasma apolipoprotein (apo) B48, B100, apoE and apoAI levels were significantly higher in the CCK-treated mice than in untreated control mice. Ligation of the bile duct, blocking CCK receptors with proglumide or inhibition of Niemann-Pick C1 Like 1 transporter with ezetimibe reduced the hypercholesterolemic effect of [Thr28, Nle31]-CCK in *LDLR^−/−^* mice. These findings suggest that CCK-increased plasma cholesterol and triglycerides as a result of the reabsorption of biliary lipids from the intestine.

## Introduction

An increase in the apolipoprotein B (apoB)-carrying lipoprotein cholesterol is a risk factor for atherosclerotic cardiovascular disease [1]. The apoB-carrying lipoproteins chylomicron and very-low density lipoprotein (VLDL) are generated in the intestine and the liver, respectively. In humans, VLDL contains a single copy of apoB100, while chylomicron contains multiple copies of apoE and a single copy of the N-terminal apoB100 (called apoB48) [2]. Interestingly, a portion of the VLDL particles produced in the mouse liver contains apoB48 rather than apoB100 [3]. These circulating lipoproteins are partially metabolized by lipoprotein lipases, generating remnant particles known as chylomicron remnants and low-density lipoprotein (LDL). Chylomicron remnants and LDL are removed from the circulation by an endocytic process mediated by LDL receptors (LDLR) and LDLR-related proteins (LRP) [2]. ApoE and apoB100 are respectively responsible for the interaction of chylomicron remnants and LDL with its receptors [2]. An increase in the generation and/or a decrease in the removal of apoB-carrying lipoproteins could result in accumulation of cholesterol in the plasma, leading to hypercholesterolemia.

Cholecystokinin (CCK) has been extensively studied as a gastrointestinal hormone and neuropeptide. Its action in the gastrointestinal system aids fat digestion and absorption, and therefore leads to an increase in cholesterol uptake via chylomicrons [4]. Specifically, CCK stimulates the secretion of pancreatic amylases, proteases and lipases. Several pancreatic lipases are able to hydrolyze cholesterol esters [5], and the resulting free cholesterol and fatty acids are taken up by enterocytes. In addition, CCK stimulates the release of bile into the small intestine [4]. Bile salts form amphipathic micelles that emulsify fats to allow lipases to access lipid molecules (such as cholesterol esters), aiding in their digestion. It has been estimated that a typical western diet contains 0.4–0.6 g of cholesterol per day, of which ∼50% is absorbed by intestinal enterocytes [6]. The body compensates for this high chylomicron cholesterol intake by reducing the amount of VLDL cholesterol synthesis, thus maintaining plasma apoB-carrying lipoproteins at physiological levels.

Biliary cholesterol and bile salts can be excreted into the feces, which could also affect cholesterol homeostasis and plasma cholesterol levels. It has been estimated that 15 to 30 g of bile salts are secreted into the intestine daily, of which ∼0.5 g is excreted as bile acids into the feces [7]. Thus, an increase in bile acid excretion in the feces has been suggested as a mechanism for elimination of excess cholesterol. In addition, excretion of biliary cholesterol into the feces is another mechanism for removal cholesterol from the body. Human adults produce 400 to 800 ml of bile daily, and cholesterol makes up ∼0.3% of the bile. Thus, 1.2–2.4 g cholesterol is released from the bile into the intestine, and typically, ∼50% (*i.e.*, ∼0.6–1.2 g) of this biliary cholesterol is excreted into the feces [7]. Apparently, the absorbed and excreted amount of biliary cholesterol by the intestine is much greater than that of dietary cholesterol. CCK increases the amount of bile released into the intestine, and thereby increases the amount of either biliary cholesterol absorption or excretion, depending on the body's need for cholesterol.

As abovementioned, the stimulatory effect of CCK on dietary and biliary cholesterol absorption would likely elevate plasma cholesterol. On the other hand, if an increase in bile release to the intestine promotes the excretion of bile acids and biliary cholesterol into the feces, CCK could also lower the plasma cholesterol level. Data from this report demonstrated that an intravenous injection of [Thr28, Nle31]-CCK, a CCK8 analogue, significantly increased the plasma levels of cholesterol and triglycerides in low-density lipoprotein receptor knockout (*LDLR^−/−^*) mice. Ligation of the bile duct, blocking CCK receptors with proglumide or inhibition of Niemann-Pick C1 Like 1 (NPC1L1) transporter with ezetimibe reduced the hyperlipidemic effect of [Thr28, Nle31]-CCK. These findings suggest that CCK can increase plasma cholesterol and triglyceride levels, and that CCK-elevated plasma cholesterol and triglycerides result from reabsorption of biliary lipids from the intestine.

## Materials and Methods

### Chemicals and Reagents

[Thr28, Nle31]-cholecystokinin (CCK) (cat# 22944) was purchased from AnaSpec Inc (San Jose, CA). Proglumide (M006) was purchased from Sigma-Aldrich (St Louis, MO). Cholesterol (RAI80015) and triglyceride (RAI80008) assay kits were purchased from Modern Laboratory Services INC (Bakersfield, CA). Ezetimibe was purchased from Selleck Chemicals LLC (Houston, TX). Antibodies against mouse apolioprotein B (apoB) (sc-11795), apoE (sc-130506) and apoAI (sc-23605), as well as horseradish peroxidase-conjugated secondary antibodies (sc-2314 and sc-2020) were purchased from Santa Cruz Biotechnology (Santa Cruz, CA). A PVDF membrane was obtained from Millipore (Billerica, MA). The ECL-plus chemiluminescence reagent was purchased from GE Healthcare Healthcare–Amersham (Piscataway, NJ).

### Animals

Male wild-type C57BL and *LDLR* ^−/−^ mice were obtained from the Jackson Laboratory (Bar Harbor, ME). The *LDLR* ^−/−^ mice were generated by Ishibashi *et al.* [8] and were backcrossed to C57BL/6 for over 10 generations. After weaning, mice were fed a chow diet containing approximately 5% fat and 19% protein by weight (Harlan Teklad, Madison, WI). All procedures for handling the animals were approved by the Institutional Animal Care and Use Committee of Meharry Medical College.

The mice (about 4 months of age) were fasted overnight, and then treated with one of four different regimens. In one of the treatment regimens, wild-type and *LDLR^−/−^* mice were injected with 50 ng/kg of [Thr28, Nle31]-CCK in approximately 30 µl phosphate buffer saline (PBS) via the tail vein. In another treatment regimen, wild-type and *LDLR^−/−^* mice were fed 0.15 ml olive oil via gavage and then injected with 50 ng/kg of [Thr28, Nle31]-CCK via the tail vein. In the third treatment regimen, wild-type and *LDLR^−/−^* mice were gavage-fed 0.15 ml water. In the fourth treatment regimen, *LDLR^−/−^* mice were subjected to bile duct ligation, injected with 150 mg/kg proglumide via a tail vein, or fed with 5 mg/kg ezetimibe via gavage. The mice were intravenously injected with 50 ng/kg of CCK at 30 min after bile duct ligation, or the administration of proglumide or ezetimibe. Blood samples were collected from the inferior vena cava or retro-orbital bleeding before and at 2 h after the [Thr28, Nle31]-CCK injection. Bile duct ligation was performed as described by Uchinami et al. [9] under anesthesia with ketamine hydrochloride (100 mg/ml) at 0.8 µl/g body weight. Briefly, an abdominal midline skin incision was made, followed by a midline incision through the abdominal musculature. The intestine and the lobes of the liver were gently retracted to expose the gallbladder and duct. The common bile duct was determined by tracking the duct from the gallbladder neck to the duodenum, and then twice ligated with 5-0 silk suture [9]. The abdominal musculature and skin were closed with suture in layers, and the mice were allowed to recover on a heating pad maintained at 37°C.

### Analysis of Plasma and Lipoprotein Lipids

Plasma total cholesterol and triglycerides were measured by spectrophotometric quantification using reagents obtained from Modern Laboratory Services, Bakersfield, CA. Briefly, aliquoted plasma was mixed with triglyceride-, or cholesterol-reaction reagents in a glass microplate. The mixtures were incubated at 37°C for 10 min. The absorbance spectra were read using a Dynex microplate reader (Thermo Labsystems, Franklin, MA) with the wavelength as described by the manufacturer. Lipid concentrations were determined based on the absorbance obtained by incubation of the triglyceride and cholesterol standards provided by Modern Laboratory Services. For measurement of the cholesterol concentration in lipoproteins, 100 µl plasma obtained from the inferior vena cava blood was fractionated using a fast protein liquid chromatography (FPLC) (Äkta FPLC 900, Amersham Biosciences, Piscataway, NJ) in a buffer containing 0.15 M NaCl, 0.01 M Na_2_HPO_4_, 0.1 mM EDTA, pH 7.5, at a flow rate of 0.5 ml/min. Forty fractions (0.5 ml/fraction) were collected. A 100 µl solution from each fraction was used for measurement of cholesterol as mentioned above. The cholesterol level in various lipoproteins was calculated from the concentration in the FPLC fractions as described previously, *i.e.*, fractions 14–17 contained VLDL and chylomicrons (referred to as VLDL), fractions 18–25 contained LDL and chylomicron remnants (referred to as LDL) and fractions 26–40 contained high-density lipoprotein (HDL) [10].

### Western Blot Analysis of Apolipoproteins

Two-µl mouse plasma was resolved on a 12% SDS-PAGE gel (for separation of apoE and apoAI) or a 5/15% gradient SDS-PAGE gel (for separation of apoB). For measurement of apolipoproteins in VLDL, LDL and HDL, a 30-µl aliquot of FPLC fractions 16, 22 and 28 was resolved on SDS-PAGE gels. Proteins were transferred to a PVDF membrane (Millipore). The membrane was blocked with 5% fat-free milk in TBS-T (2.5 mM Tris, 15 mM NaCl, 0.01% Tween 20; pH 7.6), and then consequently incubated with primary and secondary antibodies, as previously described [11]. Immunoreactive bands were visualized using ECL-plus chemiluminescence reagent (GE Healthcare Healthcare–Amersham) and analyzed with a GS-700 Imaging Densitometer (Bio-Rad, Hercules, CA).

### Enzyme Immunoassay of CCK

Plasma CCK concentration was measured using an Enzyme Immunoassay Kit (RayBiotech, Norcross, GA) following the manufacturer's instructions. Briefly, the 96-well plate, which was pre-coated with anti-rabbit secondary antibody, was incubated with anti-CCK antibody for 2.5 h in room temperature. Mouse plasma (62.5 µl) was mixed with 5 µl biotinylated CCK peptide (1 ng/ml) and 182.5 µl of appropriate assay diluent provided by RayBiotech. The mixture was incubated with the antibody-coated 96-well plate overnight at 4°C. Streptavidin-horseradish peroxidase (SA-HRP) was used to catalyze a color development reaction. Absorbance was read using a Dynex microplate reader (Thermo Labsystems, Franklin, MA) with the wavelength of 450 nm. Plasma CCK concentration was determined based on the standard curve obtained by incubation of standard CCK peptide with biotinylated CCK peptide and CCK antibody.

### Statistical Analysis

For experiments using the microplate reader, the mean value for each experiment was averaged from triplicate wells on the same plate. Data are reported as the mean ± the standard error of the mean from 5 independent experiments. Differences between treatments were analyzed by Student's *t*-test or analysis of variance followed by Tukey's post-hoc test as indicated in figure legends. Statistical significance was considered when *P* was less than 0.05. Statistix software (Statistix, Tallahassee, FL) was used for statistical analysis.

## Results

### Cholecystokinin Elevates Plasma Cholesterol and Triglycerides

**Figure 1:**
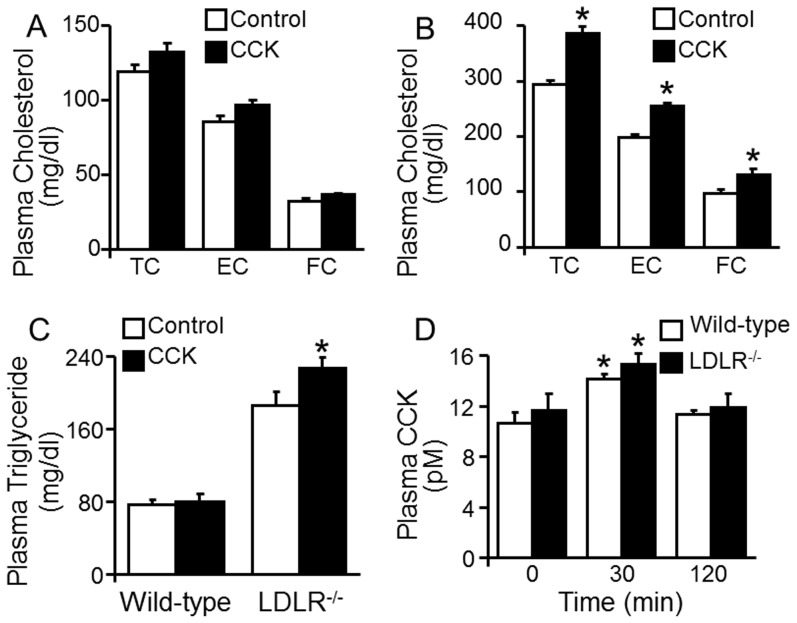
Effects of cholecystokinin on plasma cholesterol and triglycerides in fasting mice. Wild-type and *LDLR^−/−^* mice were injected with 50 ng/kg of [Thr28, Nle31]-cholecystokinin (CCK) via the tail vein. **A–C.** Blood samples were collected before (control, 0 min) and at 2 h after CCK treatment. Plasma triglycerides and total cholesterol (TC) and free cholesterol (FC) were measured colorimetrically. Esterified cholesterol (EC) was calculated as the difference between TC and FC. Values represent the mean ± SEM of 6 independent experiments. The difference between control and CCK treatment was analyzed by Student's paried *t*-test. * *P*<0.05 compared to control. **D.** Blood samples were collected before (0 time) and at 30 and 120 min after CCK treatment. Plasma CCK levels were determined using an enzyme immunoassay kit. Values represent the mean ± SEM of 4 independent experiments. The differences among time points in wild-type and *LDLR^−/−^* mice were analyzed by two-way ANOVA followed by Tukey post-hoc tests. * *P*<0.05 compared to the same genotype mice before (0 min) and at 30 or 120 min after CCK treatment.

A range of CCK doses (5 ng–75 µg/kg body weight) have been used in human and animal studies to study its biological roles [12], [13]. Our preliminary studies showed that an intravenous injection of [Thr28, Nle31]-CCK at doses of 5–50 ng/kg in 30 µl PBS induced a dose-dependent elevation in plasma cholesterol in overnight-fasted *LDLR^−/−^* mice; doses greater than 50 ng/kg did not further elevate plasma cholesterol levels. An intravenous injection of 30 µl PBS did not alter mouse plasma cholesterol or triglyceride levels in either wild-type or *LDLR^−/−^* mice (data not shown). The data in Fig. 1A–C show that plasma cholesterol and triglyceride levels were significantly higher in *LDLR^−/−^* mice than in wild-type mice. Treatment of *LDLR^−/−^*mice with CCK significantly elevated plasma cholesterol levels. Specifically, intravenous injection of 50 ng/kg of [Thr28, Nle31]-CCK increased plasma total, esterified and free cholesterol levels by 31, 28 and 26%, respectively, in *LDLR^−/−^*mice (Fig. 1B). In addition, this CCK treatment induced an ∼22% increase in plasma triglyceride in *LDLR^−/−^* mice (Fig. 1C). In contrast, the same dose of CCK non-significantly elevated plasma triglyceride and total cholesterol levels by ∼6 and 13%, respectively, in wild-type mice (Fig. 1A and 1C).

This study also determined the effect of [Thr28, Nle31]-CCK injection on mouse plasma CCK levels. Under fasting conditions, the plasma CCK concentrations before injection (time 0) were 10.7±0.9 and 11.7±1.2 pM in wild-type and *LDLR^−/−^* mice, respectively. No significant difference was observed between these two groups of mice (Fig. 1D). The CCK concentrations in these mice significantly rose ∼30%, however, 30 min after injection of 50 ng/kg [Thr28, Nle31]-CCK; values returned to basal levels when monitored 2 h after injection (Fig. 1D).

**Figure 2:**
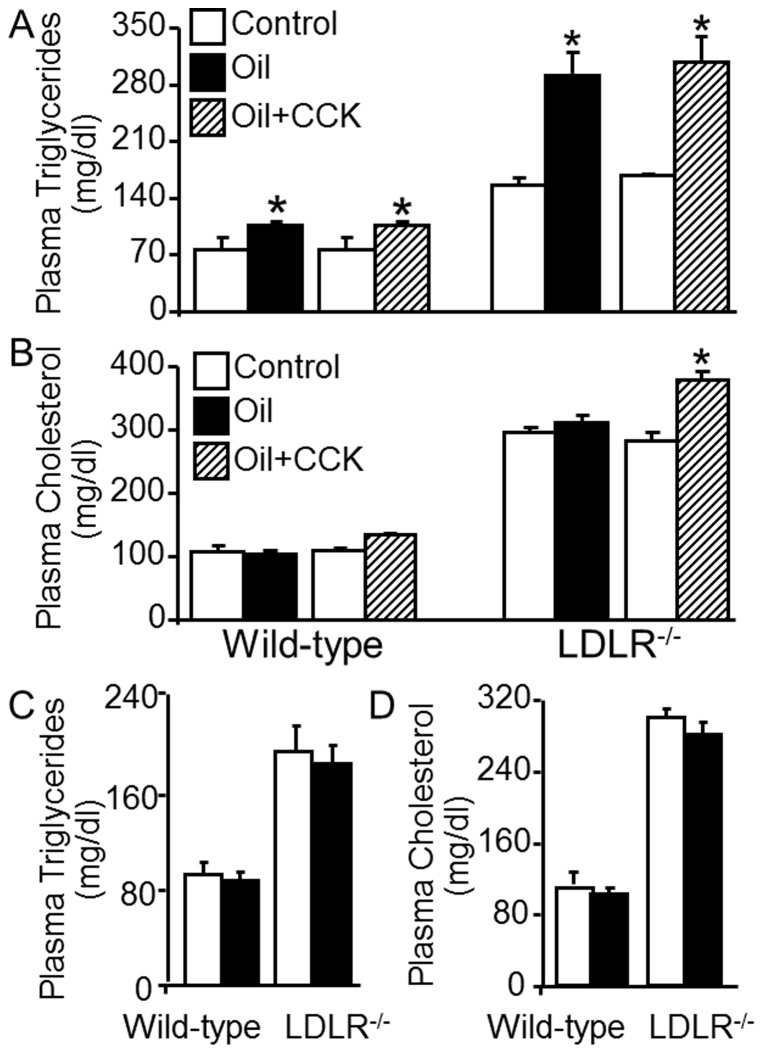
Effects of cholecystokinin on plasma cholesterol and triglycerides in oil-fed mice. **A–B.** Wild-type and *LDLR^−/−^* mice were gavage-fed with 0.15 ml olive oil and then intravenously injected 50 ng/kg of [Thr28, Nle31]-cholecystokinin (CCK). Blood samples were collected before (control) and at 2 h after the mice were treated with oil+PBS or oil and CCK. Plasma triglycerides, total cholesterol (TC) and free cholesterol (FC) were measured colorimetrically. Esterified cholesterol (EC) was calculated as the difference between TC and FC. **C.** Wild-type and *LDLR^−/−^* mice were gavage-fed with 0.15 ml water. Blood samples were collected before (control) and at 2 h after water feeding for measuring plasma TC and triglycerides. Values represent the mean ± SEM of 4–6 independent experiments. Differences among samples obtained from mice before (control) and after treated with oil+PBS or oil plus CCK were analyzed by two-way ANOVA followed by Tukey post-hoc tests (A and B). The difference between controls and water feeding was analyzed by Student's paired *t*-test (C). * *P*<0.05 compared to control.

Multiple pathways are implicated in cholesterol absorption [14]. One of them is absorption of cholesterol with chylomicrons [14]. Incorporation of absorbed triglycerides with apoB48 is an indispensable step for generation of chylomicrons [15]. To demonstrate whether an increase in triglyceride absorption enhances cholesterol absorption and whether CCK-elevated plasma cholesterol is secondary to an increased absorption of triglycerides, we studied the effect of oil feeding on plasma triglyceride and cholesterol levels in mice treated with or without CCK. It has been reported that plasma triglycerides reach a peak value around 2–3 h after feeding oil, and gradually declines thereafter [16], [17]. The data in Fig. 2A showed that orally feeding 0.15 ml olive oil alone resulted in ∼35 and 85% increase in plasma triglyceride levels, respectively, in wild-type and *LDLR^−/−^* mice at 2 h after gavage. The latter findings were significant. The data in Fig. 2A also show that treatment of wild-type and *LDLR^−/−^* mice with both olive oil and CCK did not further increase the plasma triglyceride levels when compared to treatment with oil alone. The data in Fig. 2C–D showed that oral feeding of 0.15 ml water did not alter plasma triglyceride and cholesterol levels in wild-type and *LDLR^−/−^* mice, suggesting that the elevated plasma triglycerides is a result of oil feeding rather than gavage-induced mechanic stimulation.

Data in Fig. 2B showed that oil alone did not significantly alter the plasma cholesterol level, while the combination of oil and CCK increased plasma cholesterol by 16 and 34% in wild-type and *LDLR^−/−^* mice, respectively, although only the changes in the *LDLR^−/−^* mice were significant. In fact the magnitude of the increases in cholesterol following oil plus CCK treatment in both wild-type and LDLR^−/−^ were comparable to those seen with CCK alone in these two groups of mice (Fig. 1A–B and 2B. It has been suggested that both triglycerides and cholesterol can be absorbed via chylomicrons; however, the content of these lipids in chylomicrons varies depending on the amount transported into the cells from the intestine lumen. Specifically, the contents of triglycerides and cholesterol in chylomicron particles are estimated around 85–92% and 1–3%, respectively [18]. It is highly likely that the chylomicron triglyceride content in mice fed oil is greater than that in mice not fed oil, while the cholesterol content in chylomicrons is greater in mice treated with both oil and CCK than in mice only fed oil.

### Cholecystokinin-Elevated Cholesterol is Distributed in Various Types of Lipoproteins

**Figure 3:**
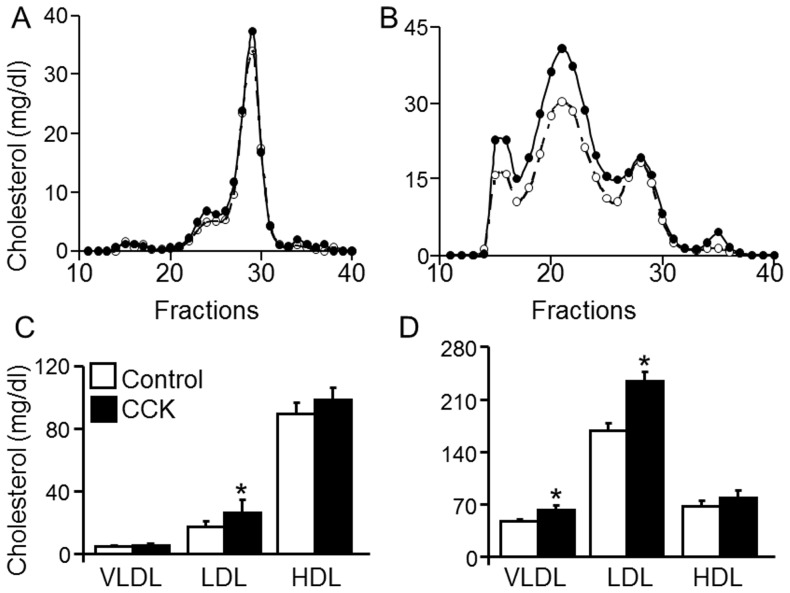
Effects of cholecystokinin on the FPLC profile of plasma cholesterol. Wild-type (**A and C**) and *LDLR^−/−^* (**B and D**) mice were intravenously injected with 50 ng/kg of cholecystokinin (CCK) or an equal volume of PBS as a control. Blood samples were collected at 2 h after CCK or vehicle injections. Mouse plasma was fractionated with a fast protein liquid chromatography (FPLC) system. Cholesterol levels in the VLDL, LDL and HDL fractions were determined with a colorimetric assay. Values represent the mean ± SEM of 5 mice per group. The difference between controls and CCK treatment was analyzed using Student's unpaired *t*-test. * *P*<0.05 compared to control.

Having established the hypercholesterolemic effect of CCK, we next investigated the distribution of the elevated cholesterol in lipoproteins. Fig. 3A and 3B illustrate typical FPLC cholesterol profiles of plasma obtained from wild-type (A and C) and *LDLR^−/−^* mice (B and D) treated with or without CCK. In the absence of CCK treatment, the plasma cholesterol was distributed mainly in HDL in wild-type mice, while *LDLR^−/−^* control mice had major increases in cholesterol associated with the LDL and VLDL fractions. These findings are consistent with previous reports [8]. The data in Fig. 3C and 3D show that CCK increased the cholesterol levels in all the lipoprotein fractions, with a greater increase in the VLDL and LDL fractions. Specifically, intravenous injection of *LDLR^−/−^* mice with 50 ng/kg of [Thr28, Nle31]-CCK resulted in 36, 45 and 21% increases in VLDL, LDL and HDL cholesterol, respectively (Fig. 3D). The same dose of CCK increased VLDL, LDL and HDL cholesterol by 9, 29 and 9%, respectively, in wild-type mice (Fig. 3C). The CCK-induced changes in wild-type mice were not statistically significant, nor was the HDL increase in *LDLR^−/−^* mice.

### Cholecystokinin Elevates Apolipoproteins

**Figure 4:**
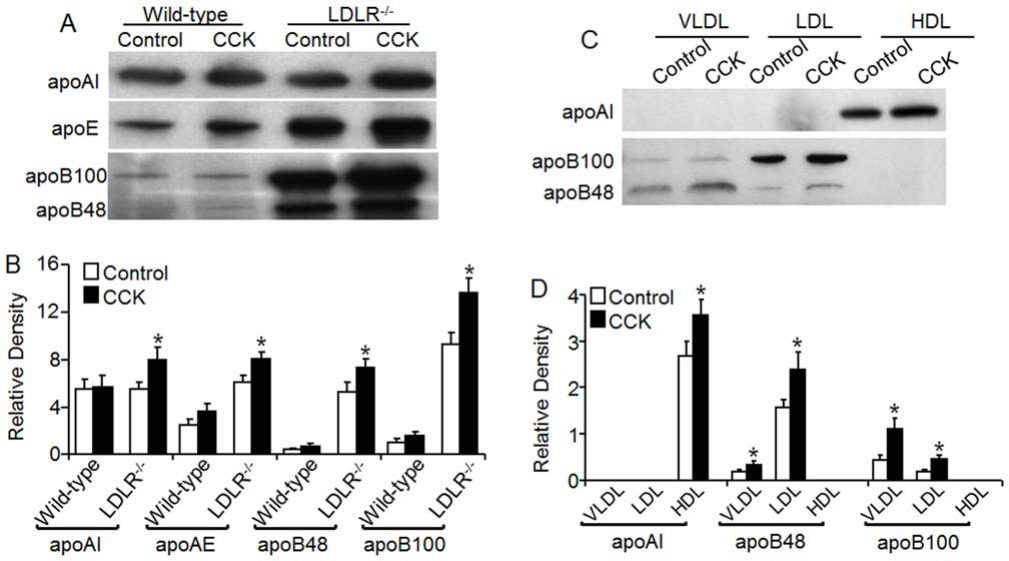
Effect of cholecystokinin on mouse plasma apolipoproteins. Wild-type and *LDLR^−/−^* mice were injected with 50 ng/kg of cholecystokinin (CCK) or an equal volume of PBS (control) via the tail vein. Blood samples were collected at 2 h after CCK or vehicle injection. **A–B.** The levels of plasma apolipoprotein (apo) AI, apo E, B100, and B48 were determined by western blot analysis. The levels of apolipoproteins were expressed as their immunoblot intensity relative to µl plasma. **C.** Mouse plasma was fractionated with a FPLC system. The apoAI, apoB48 and B100 in the VLDL, LDL and HDL fractions were determined with western blot analysis, and expressed relative to µl plasma or FPLC eluates. Values represent the mean ± SEM of four independent experiments. The difference between controlc and CCK treatment was analyzed by Student's unpaired *t*-test. * *P*<0.05 compared to control.

Having established the distribution of the increased cholesterol in lipoproteins, we then studied the impact of CCK on the plasma level of apoB, apoE and apoAI, which are the major protein components of lipoproteins. As shown in Fig. 4, plasma apoB48 and B100 were barely detectable in wild-type mice, but were significantly elevated in *LDLR^−/−^* mice, with a greatest change seen in apoB100. Intravenous injection of *LDLR^−/−^* mice with 50 ng/kg of [Thr28, Nle31]-CCK increased plasma apoB48 and B100 levels by 37 and 47%, respectively ( Fig. 4A–B ). The data in Fig. 4A–B also showed that CCK treatment increased the apoE and apoAI levels by 32 and 46%, respectively, in *LDLR^−/−^* mice. Treatment of wild-type mice with CCK slightly elevated apoB48, B100, apoE and apoAI levels; however, the difference between CCK-treated and untreated wild-type mice did not reach statistical significance (Fig. 4). We also studied the impact of CCK on the levels of apoB and apoAI in the VLDL, LDL and HDL fractions in *LDLR^−/−^* mice. As shown in Fig. 4C, apoB48 and apoB100 were distributed in the VLDL and LDL fraction, while apoAI was distributed in the HDL fraction. The data in Fig. 4C–D also showed that CCK treatment significantly elevated the VLDL/LDL marker proteins apoB48 and apoB100, and increased HDL marker protein apoAI .

### Bile Duct Ligation, Proglumide and Ezetimibe Blocked Cholecystokinin-Induced Hypercholesterolemia

**Figure 5:**
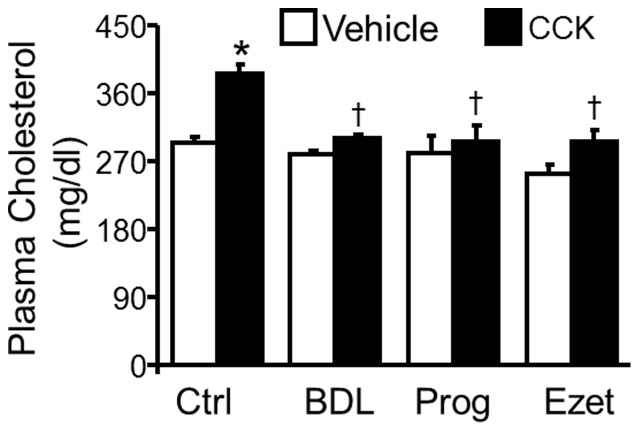
Effect of bile duct ligation, proglumide or ezetimibe on cholecystokinin-elevated plasma cholesterol. *LDLR^−/−^* mice were subjected to bile duct ligation (BDL), or intravenous injection of 150 mg/kg proglumide, or were gavage-fed with 5 mg/kg ezetimibe. At 30 min after bile duct ligation, or the administration of proglumide or ezetimibe, mice were intravenously injected with 50 ng/kg of cholecystokinin (CCK) or an equal volume of PBS (control) Blood samples were collected at 2 h after CCK or vehicle injection. Plasma cholesterol was measured with a colorimetric assay. Differences among samples obtained from mice before (control) and after treatment with CCK alone, CCK+BDL, CCK+proglumide or CCK+ezetimibe were analyzed by two-way ANOVA followed by Tukey post-hoc tests. Values represent the mean ± SEM of six independent experiments. * *P*<0.05 compared to control, and ^†^ *P*<0.05 compared to CCK treatment alone.

CCK induces cellular responses by activation of its receptors on the cell membrane. Two CCK receptor subtypes, i.e., CCK receptor-1 (CCK1R) and CCK2R, have been identified in mammalian cells [19]. CCK1R primarily is distributed in the gastrointestinal tract with lesser amounts in the central nervous system; CCK2R primarily is distributed in the central nervous system with lesser amounts in the gastrointestinal tract [19]. Proglumide has been reported to block these two CCK receptors [20]. As the data in Fig. 5 showed, pretreatment of *LDLR^−/−^* mice with proglumide almost completely blocked the hypercholesterolemic effect of CCK.

To investigate whether the hypercholesterolemic effect of CCK was related to enhanced bile release, mice were subjected to bile duct ligation. The data in Fig. 5 show that bile duct ligation reduced the CCK-elevated plasma cholesterol by 82.9% in *LDLR^−/−^* mice. These data suggest that an increase in bile release into the intestinal lumen is, at least partially, responsible for the CCK-induced hypercholesterolemia.

Transportation of cholesterol into the enterocytes by Niemann-Pick C1 Like 1 transporter (NPC1L1) is a critical step for intestinal cholesterol absorption [21]. To address the involvement of cholesterol absorption in CCK-elevated plasma cholesterol, NPC1L1 was inhibited with ezetimibe. The data in Fig. 5 show that CCK treatment did not significantly elevate plasma cholesterol inezetimibe-treated *LDLR^−/−^* mice. These findings suggest that the elevated plasma cholesterol in CCK-treated mice results from an increase in cholesterol absorption from the intestine.

## Discussion

This report, for the first time, demonstrated that [Thr28, Nle31]-CCK significantly elevated fasting plasma cholesterol in *LDLR^−/−^* mice, and that ligation of the bile duct or inhibition of NPC1L1 attenuated the cholesterol elevation effect of CCK. These observations suggest that the CCK-elevated plasma cholesterol results from absorption of intestinal cholesterol. The intestinally absorbed cholesterol could originate from multiple sources, such as diet, bile, desquamated intestinal cells and/or *de novo* synthesis within intestinal cells [15]. However, the mice used in the present study were fasted overnight. Thus the contribution of dietary cholesterol to CCK-elevated plasma cholesterol in these mice may be negligible. To the best of our knowledge, CCK does not affect *de novo* cholesterol synthesis or intestinal cell turnover. Thus, the hypercholesterolemic effect of CCK in these mice must result, at least partially, from reabsorption of biliary cholesterol. It is highly likely that CCK induces bile release into the intestinal lumen, and that enterocytes absorb biliary cholesterol back into the bloodstream, thereby elevating plasma cholesterol.

CCK induces cellular responses via its receptors. It is generally accepted that CCK1R is the predominant form in the gastrointestinal tract [19]. Existing data provide conflicting evidence regarding the impact of CCK1R on cholesterol metabolism. Specifically, null knockout of CCK1R has been shown to result in significant increases in cholesterol absorption in male mice [22]. However, in another study, male CCK1R^−/−^ and wild-type mice were fed either a normal or a fat-enriched diet for either 3 or 9 months. Experimental plasma cholesterols for both groups were available for only the 9 month data set. While there was no apparent difference in plasma cholesterol in CCK1R knockout mice versus wild-type mice fed normal chow, CCK1R knockout mice fed the fat-enriched diet had a plasma cholesterol that was ∼20% lower than that in wild-type mice (∼3.2 *vs.* ∼4 mM) [23]. It remains unknown why knockout of CCK1R increased cholesterol absorption in both mice fed a normal or lithogenic diet in one study, while in another, there appeared to be no effect of the knockout of CCK1R on plasma cholesterold in mice fed a normal diet, while there was a decrease in plasma cholesterol in the knockout mice fed a fat-enriched diet, when compared to respective wild-type controls. Further studies are required to demonstrate the CCK receptor(s) responsible for the hypercholesterolemic effect induced by injection of CCK in *LDLR^−/−^* mice.

Enterocytes deliver absorbed lipids into the circulation system via chylomicrons [21]. Data from the present study demonstrated that besides inducing hypercholesterolemia, CCK also increased the plasma level of triglycerides, another major component of chylomicrons, in fasted *LDLR^−/−^* mice. Feeding olive oil significantly increased plasma triglycerides in these mice. It is known that triglycerides in the intestine are hydrolyzed by pancreatic lipases to monoacylglycerol and fatty acids. These digested products are taken up by enterocytes and converted back to triglycerides. Together with apoB48 and cholesterol, the newly synthesized triglycerides are packaged into chylomicrons [24] which are then released from the cells, drained into the lymphatic system and ultimately discharged from the thoracic duct into the bloodstream. It has been suggested that intestinal absorption of triglycerides is a saturable process, in which transfer of triglycerides from the smooth endoplasmic reticulum membrane into the secretory pathway is rate limiting [25]. Data from this report showed that CCK did not further increase plasma triglyceride levels in oil-fed mice, as the plasma triglyceride level in mice treated with both CCK and oil was comparable to that in mice treated with oil alone.

Another finding from this report is that CCK slightly elevated plasma cholesterol and triglyceride levels in wild-type mice, although the CCK-elevated hyperlipidemia was far more moderate in wild-type compared to *LDLR^−/−^* mice. This finding suggests that the regulatory effect of CCK on plasma lipid levels is largely masked by the normal function of LDLR in wild-type mice. It is known that LDLR and LRPs mediate cellular endocytosis for removal of apoB-carrying lipoproteins from the plasma [26], [27]. The balance between the generation and removal of apoB-carrying lipoproteins is an important determinant of plasma cholesterol level. It is highly likely that the plasma cholesterol elevated by CCK under normal conditions is removed by LDLR- and LRP-mediated endocytosis of apoB-carrying lipoproteins, maintaining the plasma cholesterol at a physiological level. However, under conditions where the clearance of apoB-carrying lipoproteins is impaired, such as a LDLR deficiency, the upregulatory role of CCK on plasma cholesterol and triglycerides becomes dominant, leading to hyperlipidemia.

Evidence accumulated over the past two decades indicates that postprandial hyperlipidemia results from an increase in lipoproteins originating in both the liver and intestine, with a greater increase in those lipoproteins from the liver, namely VLDL and its remnant LDL [24]. Data from the present study demonstrated that CCK-elevated cholesterol distributed mainly in the VLDL and LDL fractions resolved from FPLC, which contain VLDL and chylomicrons, as well as their remnants [10]. In addition, we observed that CCK-induced hyperlipidemia was accompanied by increased plasma levels of both apoB48 and apoB100. This observation is consistent with the view that absorption of lipids from the intestine elevates not only plasma chylomicrons and their remnants, but also lipoproteins originating in the liver [24]. The mechanism behind this phenomenon has not been fully defined. One explanation is that the intestinal-derived chylomicrons and their remnants compete with VLDL and LDL for receptors for hepatic endocytosis [24]. The present study showed that CCK also increased plasma apoE, a protein that aids chylomicron remnant binding to LDLR and LRP for cellular endocytosis [28].

Data from the present study also showed that CCK elevated the cholesterol level in the HDL fraction, although the magnitude of the increase was not as great as seen in the VLDL/LDL fractions. In addition, CCK increased plasma apoAI levels, the major protein component of HDL. It is known that enterocytes are able to synthesize apoAI and incorporate it into chylomicrons. In the bloodstream, apoAI is transferred from chylomicrons to HDL [24]. In addition, cholesterol efflux has been suggested as a pathway for cholesterol absorption in entrocytes [14], [29]. Knockout of apoAI [14] or a deficiency of the ATP-binding cassette transporter A1 (ABCA1) [29] have reportedly reduced cholesterol absorption via this pathway. ABCA1 is a membrane protein that transfers cholesterol to apoAI to form HDL. It is possible that CCK upregulates apoAI expression and increases cholesterol efflux in enterocytes. Further studies are required to determine if this is the case.

In summary, data from this report clearly indicate that CCK is able to increase fasting plasma triglyceride and cholesterol levels in mice through a mechanism involving reabsorption of bile lipids. This CCK-induced hypercholesterolemia might be clinically important. It has been reported that bile release [30] as well as the plasma cholesterol and CCK levels [31] increase with age. It is highly possible that the age-related increase in plasma CCK is responsible for the increased bile release and hypercholesterolemia. (We thank Dr. Diana Marver for a critical reading of the manuscript. )
